# Identification of differentially expressed genes, signaling pathways and immune infiltration in rheumatoid arthritis by integrated bioinformatics analysis

**DOI:** 10.1186/s41065-020-00169-3

**Published:** 2021-01-04

**Authors:** Yanzhi Ge, Li Zhou, Zuxiang Chen, Yingying Mao, Ting Li, Peijian Tong, Letian Shan

**Affiliations:** 1 The First Affiliated Hospital, Zhejiang Chinese Medical University, Hangzhou 310053 Zhejiang, PR China; 2 Department of Epidemiology and Biostatistics, Zhejiang Chinese Medical University, Hangzhou 310053 Zhejiang, PR China; 3 The First Affiliated Hospital, College of Medicine, Zhejiang University, Hangzhou 310003 Zhejiang, PR China

**Keywords:** Rheumatoid arthritis, Bioinformatics analysis, Differentially expressed genes, Immune infiltration

## Abstract

**Background:**

The disability rate associated with rheumatoid arthritis (RA) ranks high among inflammatory joint diseases. However, the cause and potential molecular events are as yet not clear. Here, we aimed to identify differentially expressed genes (DEGs), pathways and immune infiltration involved in RA utilizing integrated bioinformatics analysis and investigating potential molecular mechanisms.

**Materials and methods:**

The expression profiles of GSE55235, GSE55457, GSE55584 and GSE77298 were downloaded from the Gene Expression Omnibus database, which contained 76 synovial membrane samples, including 49 RA samples and 27 normal controls. The microarray datasets were consolidated and DEGs were acquired and further analyzed by bioinformatics techniques. Gene ontology (GO) and Kyoto Encyclopedia of Genes and Genomes (KEGG) pathway enrichment analyses of DEGs were performed using R (version 3.6.1) software, respectively. The protein-protein interaction (PPI) network of DEGs were developed utilizing the STRING database. Finally, the CIBERSORT was used to evaluate the infiltration of immune cells in RA.

**Results:**

A total of 828 DEGs were recognized, with 758 up-regulated and 70 down-regulated. GO and KEGG pathway analyses demonstrated that these DEGs focused primarily on cytokine receptor activity and relevant signaling pathways. The 30 most firmly related genes among DEGs were identified from the PPI network. The principal component analysis showed that there was a significant difference between the two tissues in infiltration immune.

**Conclusion:**

This study shows that screening for DEGs, pathways and immune infiltration utilizing integrated bioinformatics analyses could aid in the comprehension of the molecular mechanisms involved in RA development. Besides, our study provides valuable data related to DEGs, pathways and immune infiltration of RA and may provide new insight into the understanding of molecular mechanisms.

## Introduction

Rheumatoid arthritis (RA) occurs in approximately 5 per 1000 people and can inevitably prompt severe joint damage and disability. Significant progress has been made over the past two decades with respect to the disease pathophysiology, optimal outcome measures, and effective treatment strategies, including the understanding of the comprehension in diagnosing and treating RA in the early stage [1]. The disability rate of RA ranks high among the arthritic which occurs in multiple-joint on the human body, and the incidence of this kind of arthritis is increasing year by year. The incidence of RA is occult, early diagnosis is difficult, and imaging manifestations occur comparatively late. At the point when RA is identified, the patients are usually at an advanced stage of this disease. RA would lead to multiple-joint dysfunction, disability, lower quality of life, respiratory illness, cardiovascular disease, and other comorbidities in patients not receiving intervention [2]. The etiology of RA is still ambiguous. All things considered, both genetic factors and environmental factors, contribute to the occurrence and development of RA [3].

At present, the frequently used methods for early detection and diagnosis of RA are magnetic resonance imaging, ultrasound and serological examination (including rheumatoid factor, anti-cyclic citrullinated peptide, etc). Be that as it may, there are confinements of these techniques, so the exactness and accuracy is not high [4, 5]. Computed tomography and X-rays can only detect lesions in its advanced stage, but do not detect early impairment. At the moment, the treatment of RA incorporates drug treatment, immunologic purging, functional training, surgical operation, and complementary and/or alternative medicine, and so forth [6, 7]. Therefore, it is critical to study the potential molecular mechanisms of RA synovial membrane and consequently identify more valid diagnostic techniques and more reliable molecular markers for detecting occurrence and evaluating prognosis, as well as to investigate more valid methods to control and prevent RA. Gene expression microarrays have been generally applied in studying gene expression profiles which provides a moderately new way for exploring genes and offers broad application prospects for drug-based molecular targeting. At present, large amounts of data have been published on Gene Expression Omnibus (GEO) [8]. Furthermore, integrating these databases can permit a more profound study of molecular mechanisms.

In this study, we downloaded four original microarray datasets (including GSE55235, GSE55457, GSE55584 and GSE77298) from the GEO database which incorporated a total of 76 samples, with 27 healthy controls and 49 RA samples. Differentially expressed genes (DEGs) in RA samples and control group (CG) were screened utilizing packages in R (version 3.6.1) software, and gene ontology (GO) and Kyoto Encyclopedia of Genes and Genomes (KEGG) pathways enrichment analysis of DEGs were additionally performed. After that, the protein-protein interaction (PPI) network was used to analyze the anticipated associations for a particular group of proteins through the STRING online database. Finally, the immune infiltration was analyzed by performing the CIBERSORT algorithm between RA and normal tissues, which was widely used to assess the relationship and the relative content of 22 types of immune cell subsets. Because of the limited samples and selection differences, not all the DEGs, relevant pathways and immune infiltration being screened out could be directly used as biomarkers.

## Materials and methods

### Search strategy

**Fig. 1:**
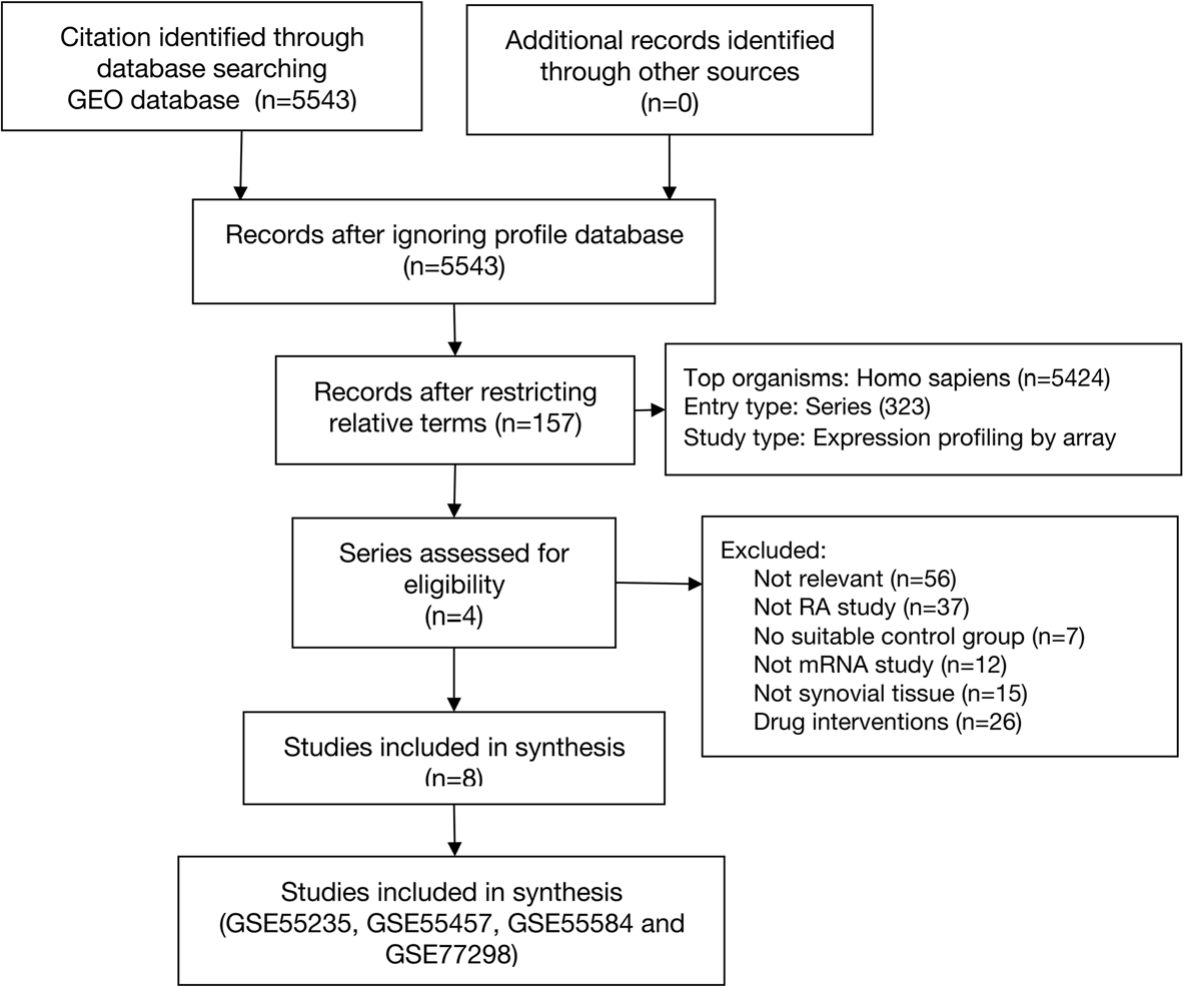
Serials selection process

We used the keyword “Rheumatoid arthritis” to search the GEO database (https://www.ncbi.nlm.nih.gov/geo/), and there was a sum of 5543 results for “Rheumatoid arthritis” in the GEO database from their inception up to November 7, 2019. By restricting the entry type (series), study type (expression profiling by array) and tissue sources (*Homo sapiens*), 5386 pieces of items that were not related to the purpose of this study were excluded. After further selection with title, summary and samples, we discovered the absence of required data in 153 items. Finally, 4 series from 2 platforms were included, and gene expression profiles of GSE55235, GSE55457, GSE55584 and GSE77298 were downloaded. Figure 1 depicted the details of the selection process.

### Microarray data information

GSE55235, GSE55457 and GSE55584 were three multi-center genome-wide transcriptomic data sets (Affymetrix HG- U133 A) from a total of 79 individuals, including 20 healthy controls, as well as 26 osteoarthritis patients and 33 RA patients. The platform for GSE77298 was GPL570, [HG-U133_Plus_2] Affymetrix Human Genome U133 Plus 2.0 Array, which included 7 synovial tissue from healthy joints and 16 synovial tissue from rheumatoid arthritis joints. Platform and series matrix file(s) were downloaded from the GEO and saved as TXT files. R software (version 3.6.1) was used to process the downloaded files.

### Integration of microarray data and DEGs

These four raw datasets were incorporated for the analysis. The coordinated microarray datasets were batch-normalized by R software (https://bioconductor.org/biocLite.R) utilizing limma packet (biocLite “limma”) analysis and then saved as a TXT file. The operating instruction codes, utilizing the R software, were processed automatically and the DEGs in CG and RA samples were analyzed by the limma package. Using R software to run the instruction code. Up or down-regulated genes were obtained independently and utilized for further analysis. The downloaded files (including platform and series of the matrix) were converted annotation package utilizing the R software.

### Data processing and identification of DEGs

Volcano plot was used to display all of the up-regulated and down-regulated DEGs using the same limma package. Then, using pheatmap package and restricting the items to 100 DEGs, the hierarchical clustering was performed. The results were visualized using a heatmap. The ID associated with the probe name was converted into gene symbol using Perl programming (https://www.perl.org) language (version 5.30.0) as well as a genome-wide annotation library (http://www.bioconductor.org/packages/release/data/annotation/html/org. Hs.eg.db.html) and then saved in a TXT file. Adjusted *P*-value<0.05 and log fold change (logFC)>2 were considered as DEGs.

#### GO and KEGG pathway enrichment analyses of DEGs

The functional and pathway enrichment of the proteins encoded by candidate genes were analyzed, and these genes were annotated using the R software. GO and KEGG pathway analysis of DEGs were performed utilizing the appropriate packages (biocLite “DOSE”, “clusterProfiler” and “pathview”). In this study, we analyzed the DEGs that were significantly up and down-regulated as determined from integrated microarray RA data, and an adjusted *P*-value of<0.05 was considered statistically significant.

### PPI network integration

The database STRING (https://string-db.org, version 11) was a precomputed worldwide resource for the exploration and analysis of interactions between known and predicted protein-protein interaction. Concerning a specific group of proteins, the network view analyzed the predicted associations. Each network node represented a different protein and the association between these nodes represented the interaction of biological molecules, which can be used for identifying interactions and associated pathways between these proteins encoded by DEGs in RA. The central nodes which were closely related to other corresponding proteins may be the core or key proteins and exert significant physiological functions.

### Immune infiltration by CIBERSORT analysis

We used the CIBERSORT [9] to analyze the normalized data filtered by Perl programming language, and immune cells infiltration matrix were acquired. In this study, twenty-two immune cells included macrophages M2, plasma cells, neutrophils, mast cells activated, T cells CD8, macrophages M1, T cells gamma delta, B cells memory, monocytes, B cells naive, T cells follicular helper, NK cells activated, dendritic cells resting, T cells CD4 memory activated, T cells CD4 naive, NK cells resting, T cells regulatory (Tregs), dendritic cells activated, eosinophils, macrophages M0, T cells CD4 memory resting and mast cells resting. The percentage of immune cells in the gene expression matrix and the relationship between two immune cells by installing “corrplot” package was calculated [10]. After that, using “ggplot2” package, the principal component analysis (PCA) was performed to discover whether there was a difference between RA and normal synovial tissues.

## Results

**Fig. 2:**
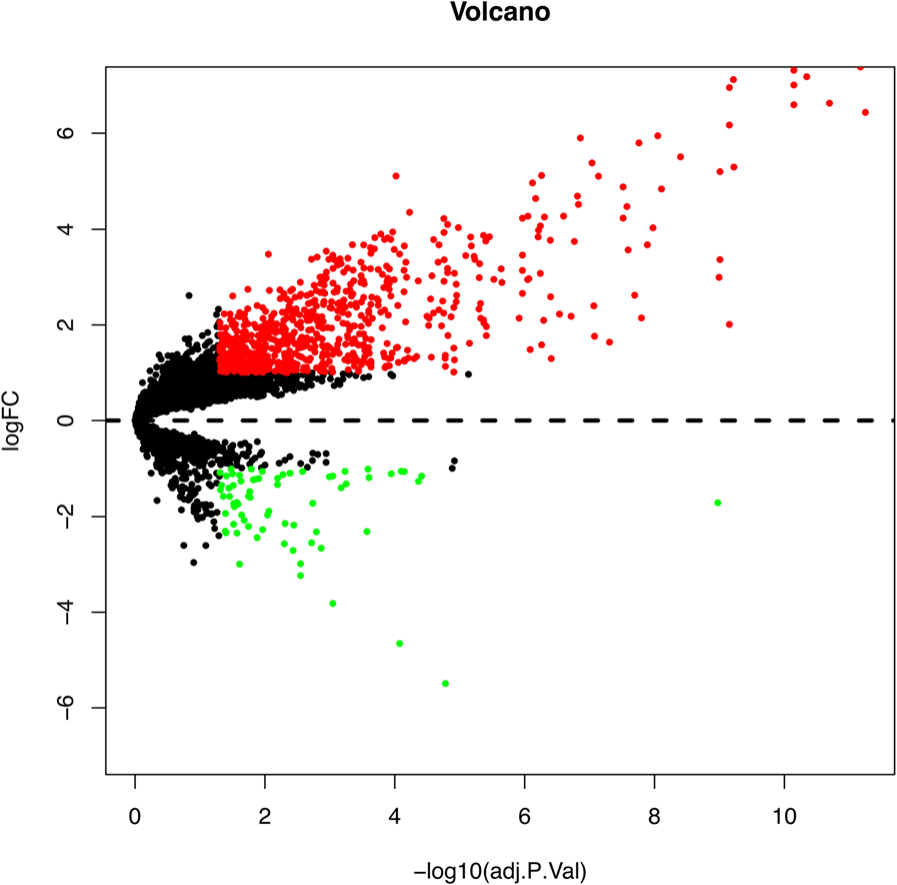
Volcano plot of the differentially expressed genes between RA and normal synovial tissues. Black points represent the adjusted *P*-value>0.05. Green points represent adjusted *P*-value<0.05 and down-regulated genes. Red points represent adjusted *P*-value<0.05 and the up-regulated genes

**Fig. 3:**
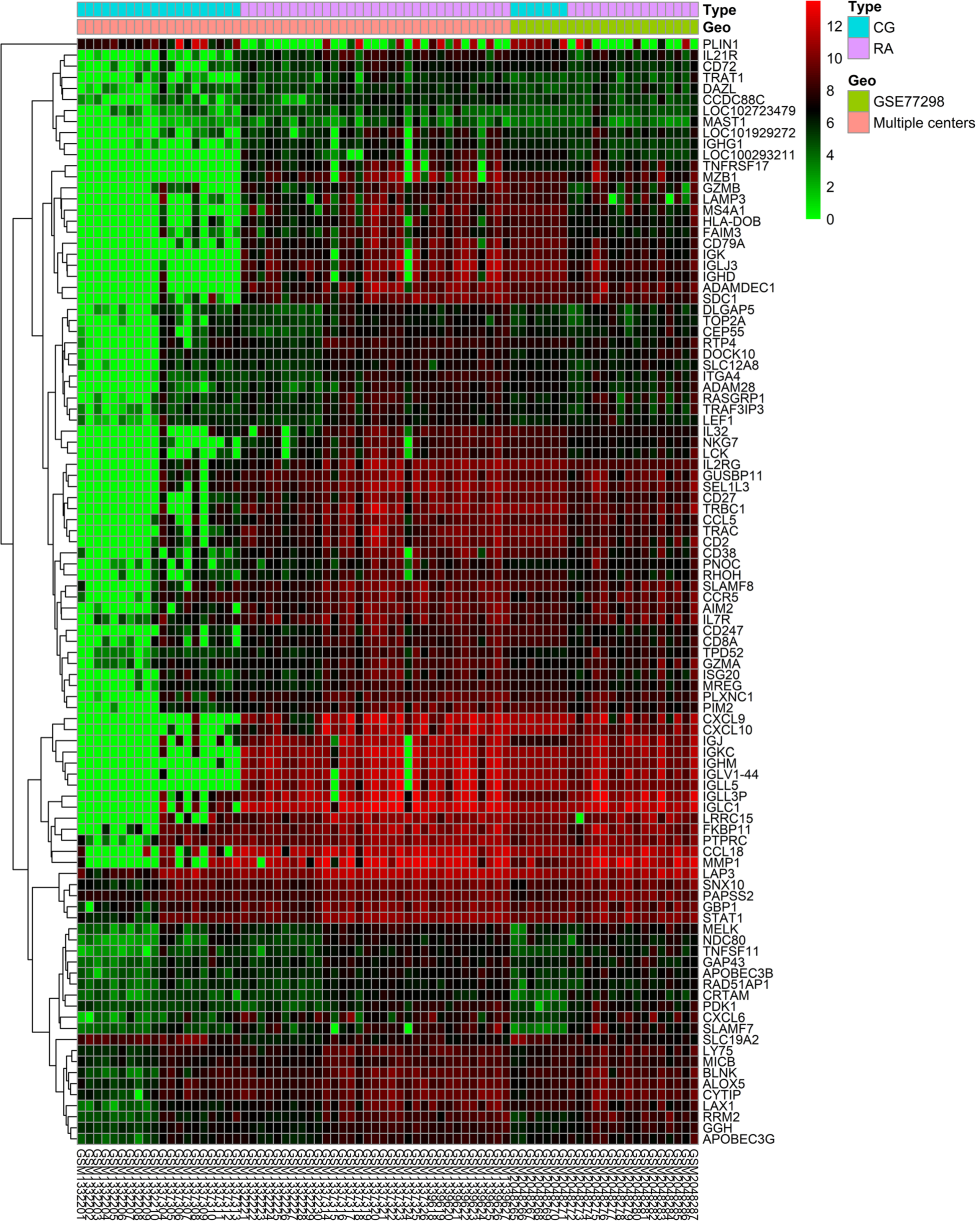
Heatmap of the top 100 DEGs according to the adjusted *P*-value and logFC. Red indicates higher gene expression and green indicates lower gene expression

An aggregate of 828 DEGs was acquired, of which 758 were up-regulated and 70 down-regulated respectively (Fig. 2). Top of the 50 up and down-DEGs from the integrated data are shown in Table 1 separately. The smaller its adjusted *P*-value, the greater possibility of DEG and higher ranking in this experiment. R-heatmap software was utilized to draw a heatmap of the 50 up and 50 down-regulated DEGs, as shown in Fig. 3.

**Table 1:** Up and down-regulated DEGs in RA by integrated data

DEGs	Gene symbol
Up-regulated	ADAMDEC1 IGHM IGJ IGKC IGLL3P IGLV1–44 IGLC1 TNFRSF17 IGLL5 CRTAM CXCL9 IGLJ3 TRAT1 SDC1 TPD52 IGK CD27 CXCL10 IL21R IGHG1 RRM2 MZB1 DAZL PNOC GUSBP11 CD79A AIM2 ALOX5 TRBC1 SNX10 LOC102723479 CCL18 MMP1 NKG7 SEL1L3 LOC101929272 CYTIP HLA-DOB TNFSF11 GGH CXCL6 PLXNC1 LCK BLNK APOBEC3B IGHD CCL5 RASGRP1 LOC100293211 SLAMF8
Down-regulated	SLC19A2 PLIN1 KLF9 ADCY2 PPAP2B EBF2 ADH1B KLF4 PPARGC1A GABARAPL1 TRHDE PHKA1 FBXW12 TCEAL2 PCK1 PCDH9 MAFF LEP RERGL SGCA ADH1C TMOD1 EDNRB ANGPTL7 ADIPOQ C6 C7 JUN ABCA8 NFIL3 CES1P1 SLC47A1 ACADL NPY1R GADD45B ATP1A2 FGF13 KCNK3 DDX3Y CLSTN2 GPC5 PODXL2 SERPINA3 TOX3 CYR61 LDB3 CNN1 CLIC5 DUOX2 FABP4

*DEGs* Differentially expressed genes, *RA* Rheumatoid arthritis

### GO term and KEGG pathway enrichment analysis of DEGs

**Fig. 4:**
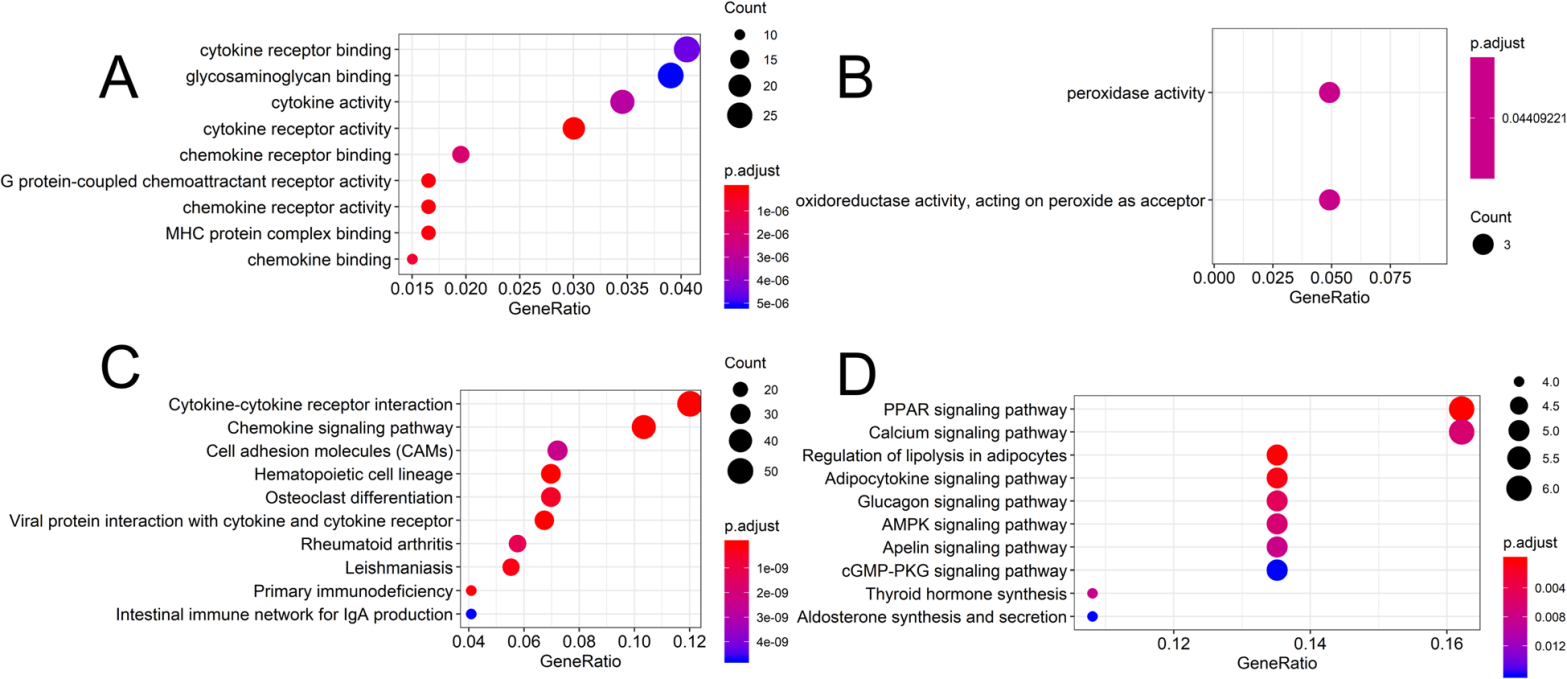
GO and KEGG pathway enrichment analysis of DEGs in GSE55235, GSE55457, GSE55584 and GSE77298. **a** GO terms in the enrichment analysis of the up-regulated genes. **b** GO terms in the enrichment analysis of the down-regulated genes. **c** KEGG terms in the enrichment analysis of the up-regulated genes. **d** KEGG terms in the enrichment analysis of the down-regulated genes

**Fig. 5:**
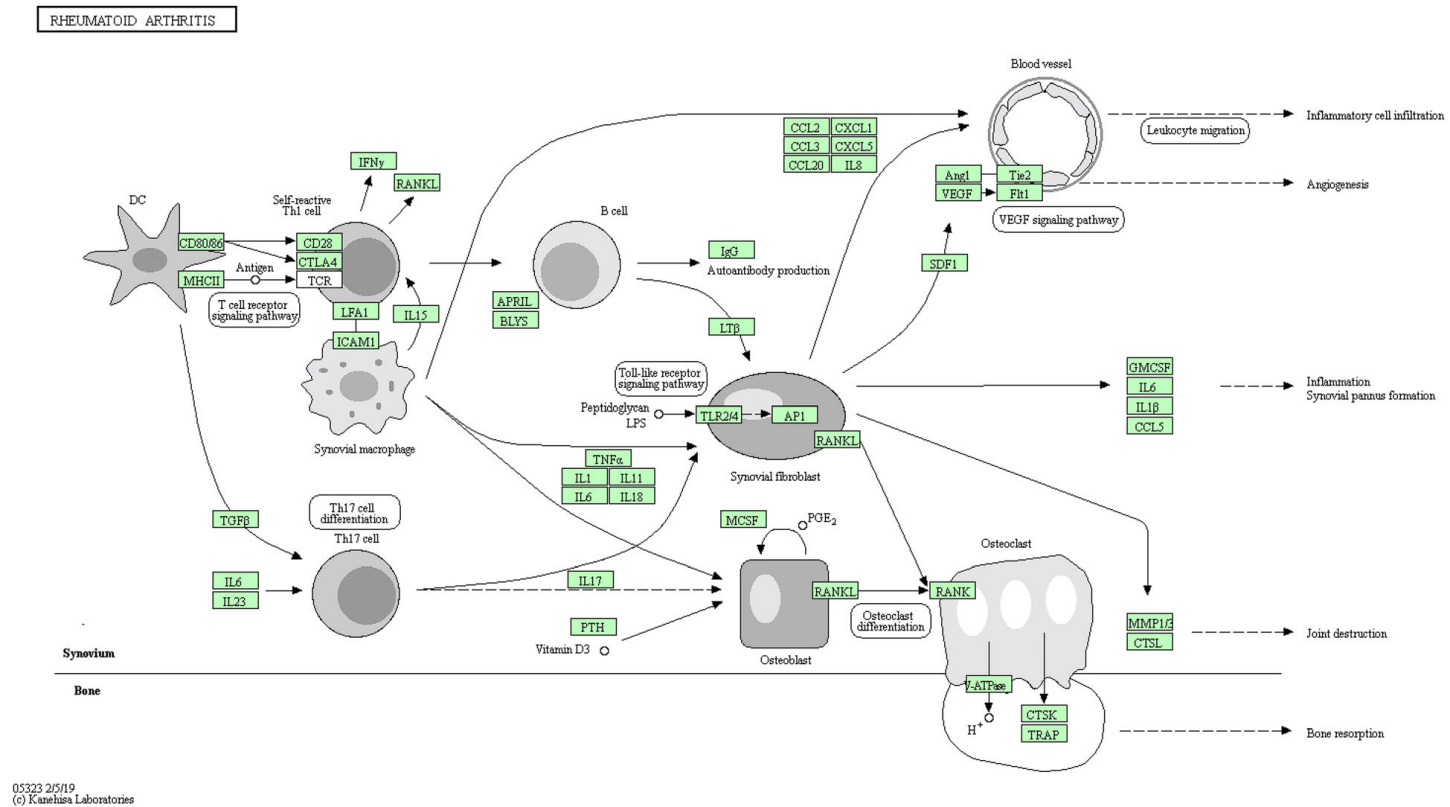
KEGG pathway enrichment analysis and pathway map for RA

The GO term and KEGG pathway enrichment analyses of up and down-regulated genes with an adjusted *P*-value of<0.05 were obtained respectively. The results of the GO term in RA were shown in Table 2 and Fig. 4 (a and b). The visual analysis results of the KEGG enrichment of DEGs in RA were shown in Table 3 and Fig. 4 (c and d). The up-regulated genes were mainly enriched in cytokine receptor activity, G protein-coupled chemoattractant receptor activity, chemokine receptor activity, MHC protein complex binding, chemokine binding, chemokine receptor binding and cytokine activity. The down-regulated genes were mostly amassed in peroxidase activity and oxidoreductase activity, acting on peroxide as acceptor. In the KEGG analysis, the up-regulated genes were mainly enriched in the chemokine signaling pathway, hematopoietic cell lineage, cytokine-cytokine receptor interaction, viral protein interaction with cytokine and cytokine receptor, primary immunodeficiency, leishmaniasis, osteoclast differentiation, rheumatoid arthritis, cell adhesion molecules (CAMs). The down-regulated enriched KEGG pathways of DEGs included PPAR signaling pathway, regulation of lipolysis in adipocytes, adipocytokine signaling pathway, glucagon signaling pathway, AMPK signaling pathway, calcium signaling pathway, thyroid hormone synthesis, apelin signaling, cGMP-PKG signaling pathway. Besides, the pathway map for targeted RA (Fig. 5) was described using in KEGG pathway enrichment. The significantly enriched terms and pathways may enlighten our minds and assist us in further study of the role of DEGs in RA.

**Table 2:** GO analysis of up-regulated and down-regulated DEGs

ID	Description	Adjusted *P*-values	Gene symbol	Count
Up-regulated				
GO:0004896	cytokine receptor activity	8.17E-09	IL21R/IL7R/IL2RG/CCR5/CCR2/CSF2RB/CCR7/CXCR4/CXCR6/CXCR3/CD4/CCR1/IL2RA/IL15RA/CX3CR1/CD74/CCRL2/IL1RL1/CXCR5/CCR6	20
GO:0001637	G protein-coupled chemoattractant receptor activity	1.30E-07	CCR5/CCR2/CCR7/CXCR4/CXCR6/CXCR3/CCR1/CX3CR1/CCRL2/CXCR5/CCR6	11
GO:0004950	chemokine receptor activity	1.30E-07	CCR5/CCR2/CCR7/CXCR4/CXCR6/CXCR3/CCR1/CX3CR1/CCRL2/CXCR5/CCR6	11
GO:0023023	MHC protein complex binding	1.30E-07	HLA-DOB/MS4A1/CD8A/HLA-DMB/HLA-DMA/KLRD1/LILRB2/LILRB1/HLA-DRA/TAPBPL/CD74	11
GO:0019956	chemokine binding	7.20E-07	ITGA4/CCR5/CCR2/CCR7/CXCR4/CXCR6/CXCR3/CCR1/CX3CR1/CCR6	10
Down-regulated				
GO:0004601	peroxidase activity	0.044092206	DUOX2/GPX3/PTGS2	3
GO:0016684	oxidoreductase activity, acting on peroxide as acceptor	0.044092206	DUOX2/GPX3/PTGS2	3

*GO* gene ontology, *DEGs* differentially expressed genes

**Table 3:** KEGG pathway of up-regulated and down-regulated DEGs

ID	Description	Adjusted *P*-values	Gene symbol	Count
Up-regulated				
hsa04062	Chemokine signaling pathway	1.90E-14	CXCL9/CXCL10/CCL18/CXCL6/CCL5/CCR5/STAT1/CCR2/ITK/PRKCB/NCF1/CXCL13/RAC2/CCR7/CXCL5/CXCL11/CXCR4/CCL13/ADCY7/CXCR6/CXCR3/DOCK2/PRKCZ/XCL1/JAK2/PRKACB/PIK3CG/CCR1/WAS/VAV1/CXCL1/PIK3CD/HCK/CX3CR1/CCL19/CCL7/CXCR5/ARRB2/GNAI2/STAT2/CCR6/FGR/PRKCD	43
hsa04640	Hematopoietic cell lineage	8.51E-13	HLA-DOB/MS4A1/IL7R/ITGA4/CD38/CD2/CD8A/CD3D/HLA-DMB/CSF1R/CD19/3ADCY7/HLA-DMA/CD3E/HLA-DPB1/CD37/HLA-DQB1/CR1/HLA-DRA/CD14/CD4/IL2RA/CSF3R/FLT3LG/CD1B/CD24/CD3G/CD7/CSF2RA	29
hsa04060	Cytokine-cytokine receptor interaction	4.76E-12	TNFRSF17/CXCL9/CD27/CXCL10/IL21R/CCL18/TNFSF11/CXCL6/CCL5/IL7R/IL2RG/CCR5/IL32/CCR2/CSF2RB/CXCL13/CSF1R/CCR7/IL15/CXCL5/IL23A/CXCL11/CXCR4/CCL13/CXCR6/CXCR3/TNFSF10/LTB/XCL1/IL27RA/TNFRSF4/CD4/CCR1/IL10RA/CXCL1/IL2RA/CSF3R/IL15RA/CX3CR1/FAS/IL18/CCL19/IFNA21/CD40LG/CCL7/TNFSF13/IL1RL1/CXCR5/CSF2RA/CCR6	50
hsa04061	Viral protein interaction with cytokine and cytokine receptor	6.00E-12	CXCL9/CXCL10/CCL18/CXCL6/CCL5/IL2RG/CCR5/CCR2/CXCL13/CSF1R/CCR7/CXCL5/CXCL11/CXCR4/CCL13/CXCR3/TNFSF10/XCL1/CCR1/IL10RA/CXCL1/IL2RA/CX3CR1/IL18/CCL19/CCL7/CXCR5/CCR6	28
hsa05340	Primary immunodeficiency	7.15E-11	CD79A/LCK/BLNK/IL7R/IL2RG/PTPRC/CD8A/CD3D/CD19/CD3E/TAP1/RFX5/ZAP70/CD4/ICOS/IGLL1/CD40LG	17
hsa05140	Leishmaniasis	1.16E-10	HLA-DOB/ITGA4/STAT1/HLA-DMB/PRKCB/NCF1/ITGB2/3ADCY7/HLA-DMA/HLA-DPB1/CYBA/CYBB/CD1B3/HLA-DQB1/CR1/HLA-DRA/PTPN6/FCGR3B/JAK2/MARCKSL1/NCF2/MAPK13/NCF4	23
hsa04380	Osteoclast differentiation	4.24E-10	TNFSF11/LCK/BLNK/STAT1/NCF1/CSF1R/FCGR2B/PLCG2/SYK/LILRB2/CYBA/LILRB4/LILRB1/CD1B3/LILRB3/FCGR3B/SIRPG/LILRA3/LCP2/PIK3CD/NCF2/MAPK13/NCF4/TREM2/TYROBP/ACP5/7CD3E8/STAT2/SPI1	29
hsa05323	Rheumatoid arthritis	1.27E-09	MMP1/HLA-DOB/TNFSF11/CXCL6/CCL5/HLA-DMB/MMP3/ITGB2/ITGAL/3ADCY7/IL15/HLA-DMA/CXCL5/IL23A/HLA-DPB1/CD86/HLA-DQB1/LTB/HLA-DRA/CXCL1/CTLA4/IL18/TNFSF13/ACP5	24
Down-regulated				
hsa03320	PPAR signaling pathway	0.000189284	PLIN1/PCK1/ADIPOQ/ACADL/FABP4/LPL	6
hsa04923	Regulation of lipolysis in adipocytes	0.000398572	PLIN1/ADCY2/NPY1R/FABP4/PTGS2	5
hsa04920	Adipocytokine signaling pathway	0.000818815	ADCY291/PCK1/LEP/ADIPOQ/ACACB	5
hsa04922	Glucagon signaling pathway	0.004887657	ADCY2/ADCY291/PHKA1/PCK1/ACACB	5
hsa04152	AMPK signaling pathway	0.006451484	ADCY291/PCK1/LEP/ADIPOQ/ACACB	5
hsa04020	Calcium signaling pathway	0.006451484	ADCY2/PHKA1/EDNRB/PLN/TNNC2/AGTR1	6
hsa04918	Thyroid hormone synthesis	0.008113772	ADCY2/ATP1A2/DUOX2/GPX3	4
hsa04371	Apelin signaling pathway	0.008113772	PLIN1/ADCY2/ADCY291/23710/AGTR1	5
hsa04022	cGMP-PKG signaling pathway	0.016012172	ADCY2/EDNRB/ATP1A2/PLN/AGTR1	5
hsa04925	Aldosterone synthesis and secretion	0.016012172	ADCY2/ATP1A2/KCNK3/AGTR1	4

*KEGG* Kyoto Encyclopedia of Genes and Genomes, *DEGs* differentially expressed genes

### Analyzing DEGs in RA using a PPI network

**Fig. 6:**
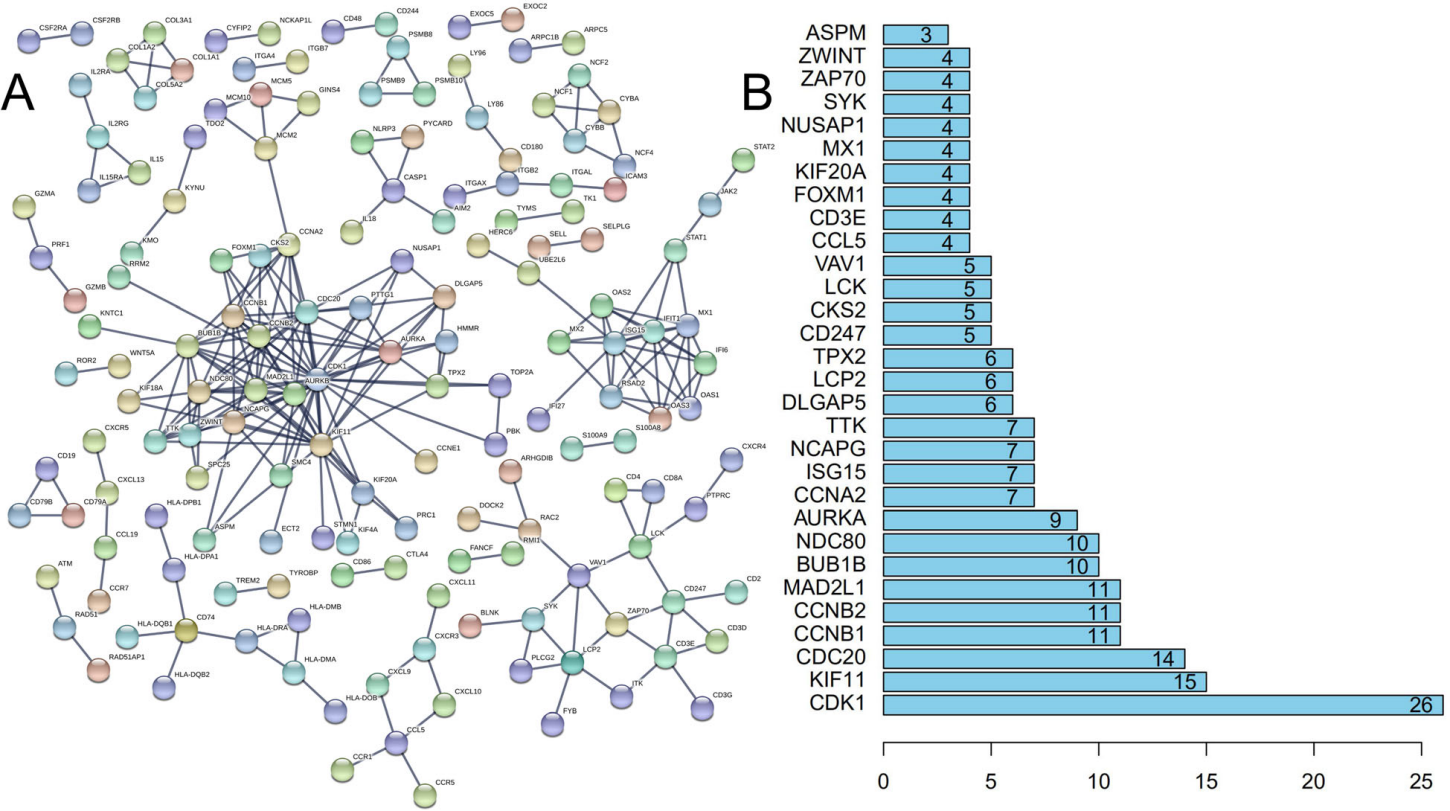
**a** PPI network (828 DEGs filtered into the PPI network that contained 103 nodes and 168 edges). **b** The predicted association rank (from low to high) of the top 30 genes in the PPI network

The DEG expression products in RA were constructed by way of the STRING database to construct PPI networks (minimum required interaction score: 0.990). After deleting all isolated and partially disconnected nodes, an integrated network was built, as shown in Fig. 6a. The 30 most significant genes (Fig. 6b) which had been displaying statistical significant interaction were CDK1, KIF11, CDC20, CCNB1, CCNB2, MAD2L1, BUB1B, NDC80, AURKA, CCNA2, ISG15, NCAPG, TTK, DLGAP5, LCP2, TPX2, CD247, CKS2, LCK, VAV1, CCL5, CD3E, FOXM1, KIF20A, MX1, NUSAP1, SYK, ZAP70, ZWINT and ASPM. Among these genes, CDK1, KIF11, and CDC20 possessed the highest node degree.

### Immune infiltration analyses

**Fig. 7:**
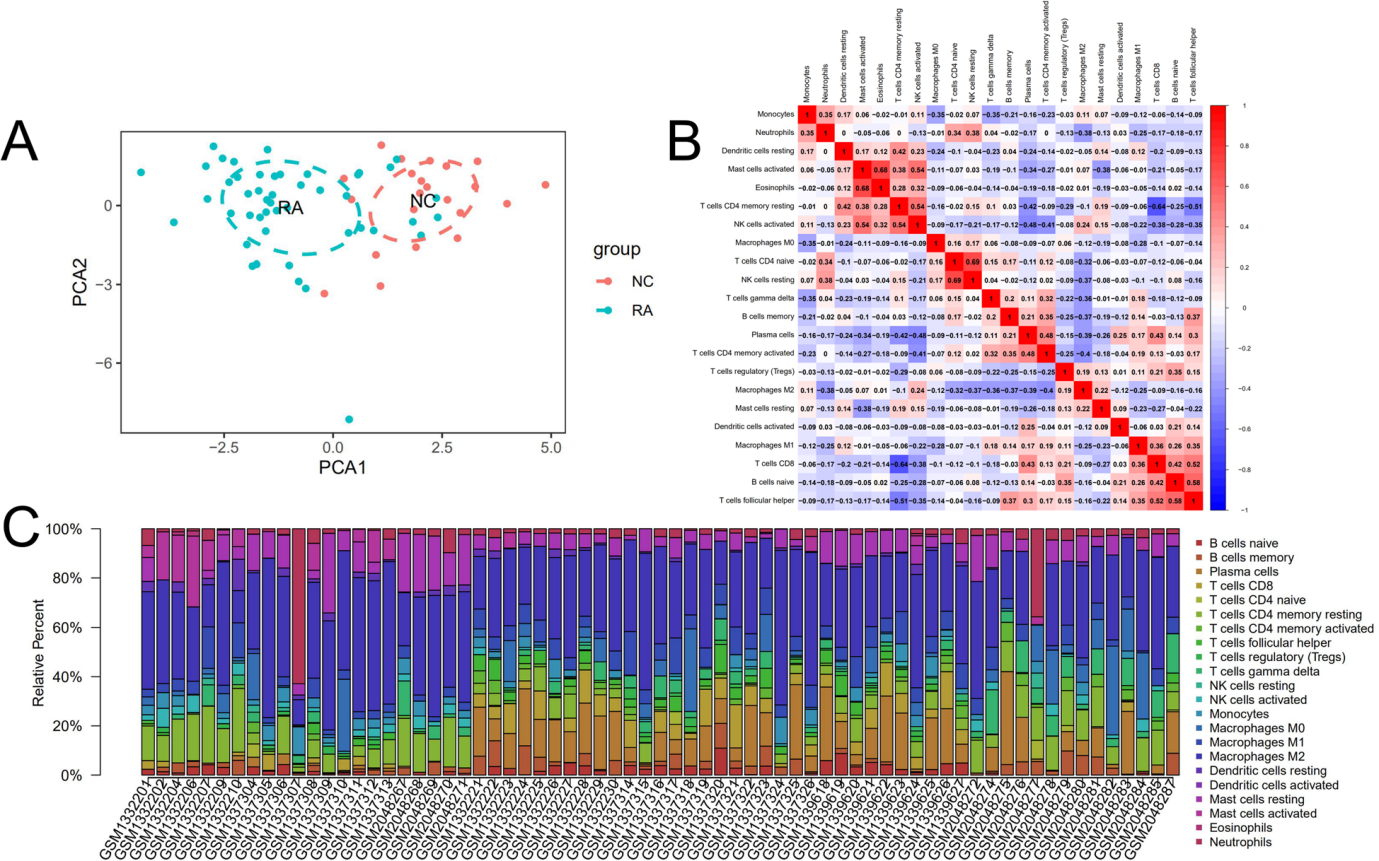
Results of CIBERSORT analysis of Gene Expression Omnibus database. **a** Principal component analysis (PCA) was performed on two groups. Red points and ellipse indicate RA sample, and green points and ellipse indicate normal samples. **b** Correlation matrix of infiltration degree of immune cells in RA samples. Red indicates trends consistent with the positive correlation and blue indicates trends consistent with the negative correlation between two immune cells. The bigger size of the numbers statistics data represents the more positive or negative correlation. **c** Landscape of immune cell infiltration

**Fig. 8:**
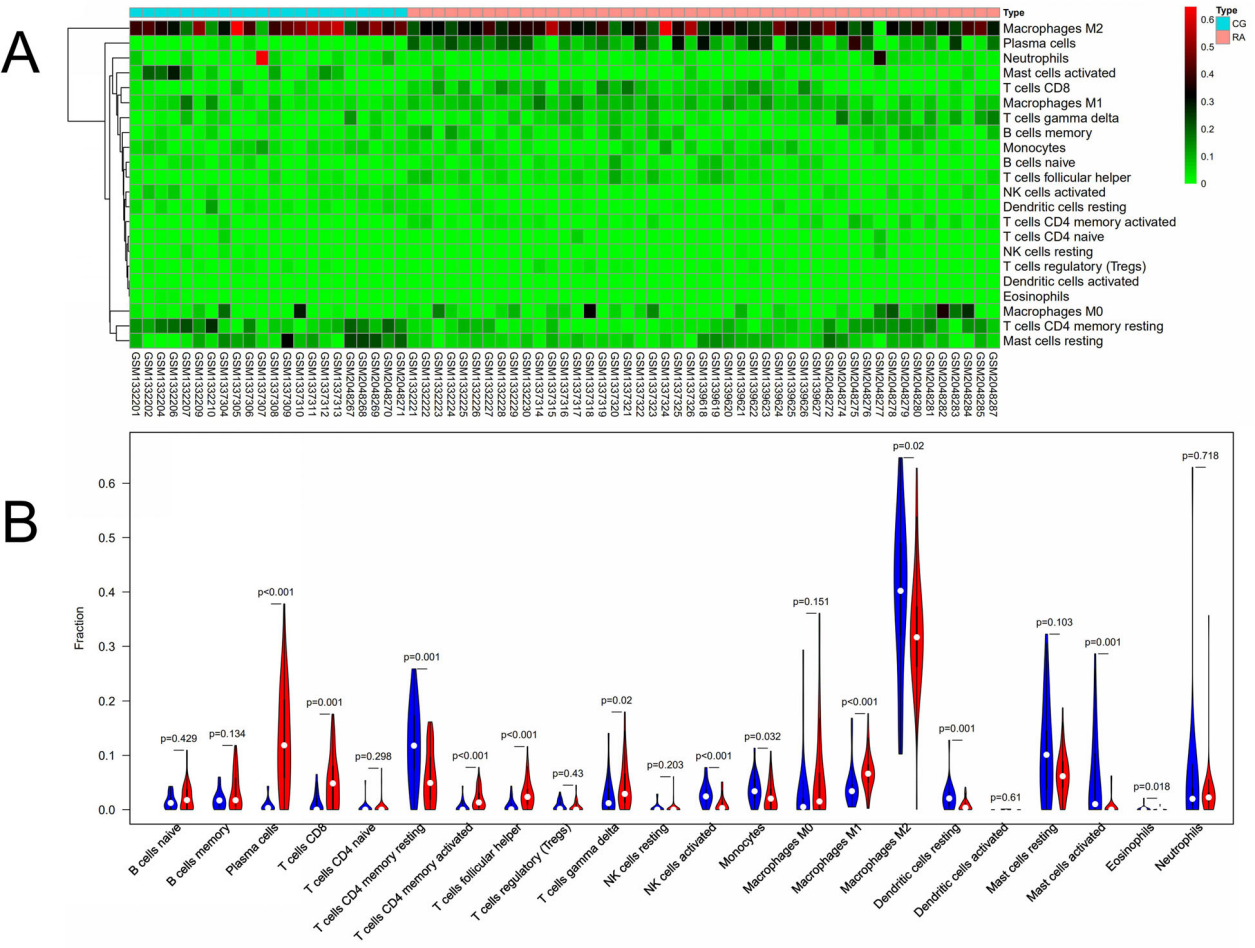
The landscape of immune infiltration between RA and normal controls. **a** The distribution of 22 immune cells in 69 filtered gene matrix. Red indicates higher immune infiltration expression and green indicates lower expression. **b** Violin diagram of immune cell proportions in two groups. The blue fusiform fractions on the left represent the normal group and the red fusiform fractions on the right represent the RA group.

PCA depicted no overlap of these two elliptical clusters and showed that there was a significant difference (*P*<0.05) in immune cell infiltration between the RA group and the healthy control group (Fig. 7a). The corheatmap (Fig. 7b) result showed that mast cells activated and eosinophils had a positive correlation (value = 0.68). T cells CD8 had a significant negative correlation with T cells CD4 memory resting (value = − 0.64). Correlation heatmap (Fig. 7c) summarized the results obtained from 69 filtered gene expression matrix and the relative percent of the 22 immune cells was shown in Fig. 8a. Compared with normal tissue, the violin plot of the immune cell showed that, plasma cells, T cells CD8, T cells CD4 memory activated, T cells follicular helper, T cells gamma delta and macrophages M1 infiltrated statistically more, while T cells CD4 memory resting, NK cells activated, monocytes, macrophages M2, dendritic cells resting, mast cells activated and eosinophils infiltrated statistically less in RA tissue (Fig. 8b).

## Discussion

The characteristics of RA is synovitis, systemic inflammation, and the arrival of autoantibodies [2]. As a result, the synovial membrane breaks down the body’s immune system, causing chronic inflammation, destruction of cartilage and bone, and dysfunction to other essential organs [11–13]. It is reported that 50% of the risk for occurrence and development of RA is related to genetic factors. At the same time, smoking is an environmental risk factor for RA. The early onset of RA is not easy to identify, and in the meantime, cartilage and bone disintegration are frequently found in the end stages of this disease. RA occurrence and development can occur at any age, gender, and nationality for complex biological processes, and the positive rate of serum examination is low as well as non-specific. Consequently, it is miles critical to observe and study the mechanisms and development of RA at the molecular level. Based on this, differentially expressed genes (DEGs) have been efficaciously used to predict the response of therapeutic approaches for RA patients. As an example, the capability of certain genes (type I interferon-responsive) to predict the nonresponder of rituximab [14] and anti-tumor necrosis factor [15].

In this study, we integrated gene expression profile datasets from four specific groups (GSE55235, GSE55457, GSE55584 and GSE77298) and used R (version 3.6.1) to analyze these datasets. A total of 828 DEGs were identified using the limma package, consisting of 758 up-regulated genes and 70 down-regulated genes. The pinnacle 20 most significantly up-regulated genes were ADAMDEC1, IGHM, IGJ, IGKC, IGLL3, IGLV1–44, IGLC1, TNFRSF17, IGLL5, CRTAM, CXCL9, IGLJ3, TRAT1, SDC1, TPD52, IGK, CD27, CXCL10, IL21R and IGHG1. The pinnacle 20 most significantly down-regulated genes were SLC19A2, PLIN1, KLF9, ADCY2, PPAP2B, EBF2, ADH1B, KLF4, PPARGC1A, GABARAPL1, TRHDE, PHKA1, FBXW12, TCEAL2, PCK1, PCDH9, MAFF, LEP, RERGL and SGCA. Constructing a PPI network (minimum required interaction score = 0.990) of DEG-encoding proteins from the STRING database and screening the 30 most significant related genes. The enriched GO of DEGs in RA were analyzed by R software, and correlation analysis confirmed that the up-regulated genes have been specifically involved in cytokine receptor activity, G protein-coupled chemoattractant receptor activity, chemokine receptor activity, MHC protein complex binding, chemokine binding, chemokine receptor binding and cytokine activity, and that the down-regulated DEGs were mainly involved in peroxidase activity and oxidoreductase activity, acting on peroxide as acceptor. This finding is consistent with the knowledge that cytokine, chemokine and peroxidase activity play crucial roles in the RA occurrence and progression.

The detection of autoantibodies (including RA, A-CCP, CRP) in RA patients is an identification that distinguishes the disease from other inflammatory arthritis, such as psoriatic arthritis, reactive arthritis and osteoarthritis. In addition to the clinical symptoms and signs arising from arthritis processes in the joints, muscle weakness around joints is also commonly reported by RA patients [16–18]. Takashi Yamada et al. [18] discovered that altered Ca^2+^ and free radical signaling (such as reactive oxygen and reactive nitrogen species) can result in RA-based muscle weakness. In a general way, RA with CCP + RF+ subjects had excessively high citrulline-specific IgG binding, and CCP + RF- and CCP-RF+ subjects had modest binding to array peptides [19]. As a systemic autoimmune disease, RA is characterized by inflammation and angiogenesis in the synovium. Many cytokines and inflammatory medium are observed in synovial tissues and synovial fluids, whose function is to display angiogenic properties. Inhibitor of DNA binding 1, one of the transcription factors, is a marker of cellular self-renewal. This factor within the bone marrow causes a significant reduction of endothelial progenitor cell association with tumor-related vasculogenesis [20, 21]. Amélie Simon et al. [22] observed that microscopic polyangiitis is vasculitides typical of necrotizing inflammation for small-sized vessels and is usually connected with serum positivity for those anti-neutrophil cytoplasmic antibodies. In most conditions, anti-neutrophil cytoplasmic antibodies are directed against two constituents of neutrophil primary granules as well as monocyte lysosomes: myeloperoxidase or proteinase 3.

Furthermore, the up-regulated enriched Kyoto Encyclopedia of Genes and (KEGG) pathways of DEGs included the chemokine signaling pathway, hematopoietic cell lineage, cytokine-cytokine receptor interaction, viral protein interaction with cytokine and cytokine receptor, primary immunodeficiency, leishmaniasis, osteoclast differentiation, rheumatoid arthritis, cell adhesion molecules (CAMs). The down-regulated enriched KEGG pathways of DEGs included PPAR signaling pathway, regulation of lipolysis in adipocytes, adipocytokine signaling pathway, glucagon signaling pathway, AMPK signaling pathway, calcium signaling pathway, thyroid hormone synthesis, apelin signaling, cGMP-PKG signaling pathway. Relative studies have demonstrated that fibroblast-like synoviocytes play a crucial role by producing cytokines in all stages of RA. Once fibroblast-like synoviocytes are activated during RA, a series of inflammatory factors and proteases will be produced involved in the inflammatory response, causing progressive destruction of bone and cartilage [23].

RA is associated with an increase in mortality. Previous research displayed that the occurrence rate of the malignancies in RA patients has been reported to be high [6]. A review of scientific studies compiled in Romania demonstrated findings that anemia and other chronic disease manifestations are relatively common in approximately 6–10% of RA patients, and are all related to worse outcomes in particular functional impairment and mortality [24, 25]. The adaptive immune system is closely connected with the generation of the anti-tumor immune response. For that reason, RA patients with gastrointestinal cancer history must be carefully monitored while receiving the treatment of disease-modifying antirheumatic drugs [26]. However, in many factors, tumor necrosis factor-α is recognized as performing biological functions associated with the pathogenesis of RA [27]. Its capabilities include chemokine amplification, endothelial cell activation, leukocyte accumulation [28], experiencing cardiovascular comorbidity [29], acceleration destruction of osteoclast and chondrocyte, and demonstrating metabolic syndrome [30]. Related studies have reported that peroxisome proliferator-activated receptor (PPAR)-γ may additionally induce activation Wnt/β-catenin signaling [31]. Numerous studies have indicated that decreased expression of adipocyte genes such as nuclear receptors PPARg in the RA synovial tissue [32, 33], and PPARg mediates mesenchymal stem cells as well as fibroblast-like synovial cells differentiation into adipocytes [34]. PPAR-γ activators performed significantly anti-inflammatory and anti-degeneration roles in rheumatoid arthritis [35]. According to a study of adjuvant-induced arthritis in rats’ synovium, inhibition PPAR-γ expression by T0070907 or PPAR-γ siRNA could significantly promote the proliferation of fibroblast-like synoviocytes and expressions of c-Myc, Cyclin D1, MMP-1, and MMP-9, except for TIPM-1 [31, 36]. Meanwhile, compared with normal tissues, PPAR-γ was obviously reduced whether immunohistochemical technique or protein detection by western blot [36]. A lot of experimental results were commendably consistent with the result in this integrated study and suggested that PPAR-γ might play a pivotal role during RA synovial tissue activation. As for the gene expression of AMPK in those newly diagnosed RA patients, a master regulator of the metabolic process was decreased in the peripheral blood leukocytes and elevated levels of TGF-β1 in plasma accounts for the occurrence of RA pathogenesis [37]. Recent data evidence suggested that S100A8/A9 was a member of the Ca^2+^ binding S100 protein family and had become a hot topic as a critical alarmin modulating the inflammatory response. Using small-molecule inhibitors that block off S100A8/A9 activity can exhibit beneficial functions on disease relative activities in animal models of autoimmune diseases such as RA [38, 39].

We constructed a PPI network of the protein encoded by DEGs and identified the subsequent top 10 closely related genes: CDK1, KIF11, CDC20, CCNB1, CCNB2, MAD2L1, BUB1B, NDC80, AURKA and CCNA2. These genes are key nodes for the construction a PPI network and play a distinct role in the pathogenesis of RA. In accordance with the proinflammatory CDK signaling, p16^INK4A^ protein as a Cyclin-Dependent Kinases inhibitor in synovial fibroblasts also demonstrated an inhibitory action in the development of RA [40]. Ectopic expression of p16^INK4A^ protein can also suppress LPS-induced IL-6 expression in macrophages [41], and simultaneously enhance the observations that CDK inhibitory proteins relative features to counteract inflammation [42]. Interleukin-6 (IL-6) signaling was a critical target in inflammatory pathways [43]. In patients with RA, the high level of IL-6 and IL-6R are found in both serum and synovial fluid of related joints affected by the disease. IL-6 is a cytokine serving several biological and biochemical functions that affect the immune and vasculature system. Generally speaking, conventional IL-6 signaling is in charge of the anti-inflammatory capabilities of IL-6, conversely, trans-signaling is in charge of the pro-inflammatory properties of IL-6. Consequently, disorders of the IL-6 axis can result in the onset or progression of disease states, especially in autoimmune and inflammatory dysregulation [44]. Activation of epidermal growth factor receptor (EGFR) signaling leads to the propagation and metabolism of synovial fibroblasts in RA. Beyond that, in addition to its function in propagation and metabolism, EGFR can generate cytokine in synovial tissues during the pathogenesis of RA. Some animal experiments have yielded potentially prospective results aiming at target EGFR involving RA. As a result, pharmacologic modulations or its ligands targeting EGFR may reveal undiscovered methods for the treatment of RA [45]. EGF receptor is a tyrosine kinase. At present, only NEK6 and CDK1 kinases can phosphorylate KIF11 at Ser1033 and Thr926 respectively, causing the combination of microtubules and KIF11 in the process of mitotic spindle assembly [46–48]. Some inflammatory cytokines are controlled by the expression of the c-Fos. Both IL-1β and c-Fos are interacted with each other, including its gene expression and activities, and causing a cross-linking effect that is a vital mechanism to arthritic joint destruction. As a result, the blockade of IL-1β, c-Fos, or link between both can be effective therapeutically as a treatment method for RA patients’ joint destruction [49]. Researchers at the University of Chicago found through mice experiments that the inhibition of c-Myc or c-Raf-1 can significantly decrease the invasiveness of RA synovial fibroblasts. Besides, dominant-negative mutants c-Raf-1 reduced the expression of phosphorylated c-Jun in vivo as well as the expression of disease-relevant MMPs [50].

In the current study, using CIBERSORT analytical tool, the relative percent and content of 22 immune cell subsets, what we have performed, were the most comprehensive analysis in RA synovial tissues to date. We found that the proportion of mast cells activated, eosinophils and T cells CD4 memory resting was high in the normal synovial tissue, while T cells CD8 accounted for RA synovial high expression. A study has found that T cells, particularly CD8 infiltration in the synovial tissues, were considered as predictors of RA development and the presence of antibodies against citrullinated peptides [51]. In the correlation analysis of the infiltration degree of immune cells, CD8+ T cells were negatively correlated with the infiltration degree of resting CD4+ memory T cells, which meant the binding of CD4 + T cells and RA synovial antigen in the microenvironment was decreased [52].

In the last few years, DEGs identification and further bioinformatics analysis was performed and these results may provide new perspective for the study of RA. However, a limitation of this study was not experimentally validated, which may need to perform in the future studies.

## Conclusions

It is beneficial for the research community to study this network to further examine and understand the interaction between RA relevant DEGs. These findings may help us to enhance our general understanding of the molecular mechanism of RA. However, further relevant molecular biological experiments are required to affirm the function of the identified genes associated with RA.

## Data Availability

The datasets used and analyzed during the current study are available from the corresponding author on reasonable request.

## Abbreviations

DEGs: Differentially expressed genes
RA: Rheumatoid arthritis
GO: Gene ontology
KEGG: Kyoto Encyclopedia of Genes and Genomes
logFC: Log fold change
PPI: Protein-protein interaction
PCA: Principal component analysis
CG: Control group
PPAR: Peroxisome proliferator-activated receptor
IL-6: Interleukin-6
EGFR: Epidermal growth factor receptor
